# Anti-cancer activity of 7-methoxyheptaphylline from *Clausena harmandiana* against PANC-1 pancreatic cancer cells and its sustainable extraction method

**DOI:** 10.1371/journal.pone.0334901

**Published:** 2025-10-16

**Authors:** Juthamart Maneenet, Suresh Awale, Chantana Boonyarat, Pornthip Waiwut, Rawiwun Kaewamatawong

**Affiliations:** 1 Natural Drug Discovery Laboratory, Institute of Natural Medicine, University of Toyama, Japan; 2 Faculty of Pharmaceutical Sciences, Khon Kaen University, Khon Kaen, Thailand; 3 Faculty of Pharmaceutical Sciences, Ubon Ratchathani University, Warin Chamrap, Ubon Ratchathani, Thailand; Kerman University of Medical Sciences, IRAN, ISLAMIC REPUBLIC OF

## Abstract

Pancreatic cancer presents a significant therapeutic challenge characterized by poor survival rates, leading to development of innovative treatment strategies. This study evaluated the anti-cancer potential of 7-methoxyheptaphylline (7-MH), a carbazole alkaloid from *Clausena harmandiana*, against pancreatic cancer cells (PANC-1) and developed an environmentally sustainable extraction methodology using ultrasonic-assisted extraction (UAE). 7-MH demonstrated selective cytotoxicity against PANC-1 cells with preferential activity under nutrient deprivation conditions (PC_50_ = 4.54 μM) compared to nutrient-rich conditions (IC_50_ = 46.84 μM). This compound exhibited minimal toxicity toward normal MCE301 epithelial cells (IC_50_ = 83.4 μM). Live-cell imaging showed dose-dependent apoptotic morphology including membrane blebbing and cell shrinkage within 24 hours. At concentrations of 25 and 50 μM, the compound significantly inhibited wound closure and colony formation, suggesting antimetastatic properties. Mechanistic analysis exhibited that 7-MH suppressed the phosphatidylinositol 3-kinase (PI3K)/protein kinase B (Akt)/mammalian target of rapamycin (mTOR) signaling pathway specially under nutrient deprived conditions. Western blot analysis showed 45% reduction in Akt expression, 43% decrease in mTOR phosphorylation, and complete inhibition of Akt phosphorylation at 20 µM concentration. For sustainable extraction of 7-MH, UAE using ethanol was optimized through response surface methodology (RSM) with central composite design (CCD). The optimal protocol (50 °C, 60 minutes, 0.40 g/10 mL plant/solvent ratio) achieved 1.26 ± 0.02% yield with only 3.55% deviation from predicted values. This developed extraction method provides an efficient and environmentally sustainable alternative to conventional halogenated solvent extraction methods. These findings offer valuable insights for advancing natural product-based cancer therapeutics and demonstrate the implementation of sustainable natural products extraction principles.

## Introduction

Pancreatic cancer is one of the most lethal malignancies globally, characterized by an extremely poor five-year survival rate. Ranking seventh in global cancer mortality, this disease is projected to become the second-deadliest cancer in the United States by 2030 [1,2]. This devastating disease has the poorest prognosis among cancers, primarily due to its asymptomatic progression during early stages, leading to late-stage diagnoses when conventional interventions are no longer curative [3]. A distinctive characteristic of pancreatic tumors is their hypovascular nature, which creates a microenvironment of severe nutrient and oxygen deprivation. However, despite these harsh conditions, pancreatic cancer cells demonstrate remarkable proliferative capacity through metabolic adaptation, a phenomenon termed “austerity” in cancer research, enabling their survival in nutrient-scarce environments [4].

Among the established models for pancreatic cancer research, the PANC-1 human pancreatic carcinoma cell line has been extensively used in this field. These cells exhibit epithelial morphology and contain mutations in key oncogenes, including Kirsten rat sarcoma virus *(*KRAS), tumor protein p53 (TP53), and cyclin-dependent kinase inhibitor 2A (CDKN2A), which are frequently observed in human pancreatic cancers. The PANC-1 model provides a valuable platform for investigating therapeutic interventions under both standard and nutrient-deprived conditions that mimic the tumor microenvironment [5,6].

Natural products continue to serve as crucial sources for anticancer drug discovery. *Clausena harmandiana* (Pierre) Pierre ex Guillaumin, a member of the Rutaceae family, has been traditionally applied in northeastern Thai medicine for its anti-inflammatory properties [7]. Phytochemical investigations have revealed that the root bark of this plant contains diverse bioactive compounds, including coumarins, limonoids, and carbazole alkaloids, which exhibit anticancer, anti-inflammatory, analgesic, antimalarial, antidiabetic, antibacterial, and anti-Alzheimer’s disease properties [8–15]. Of these bioactive compounds, 7-methoxyheptaphylline (7-MH) stands out as a promising carbazole alkaloid. Previous studies have demonstrated its significant cytotoxic activity against multiple cancer cell lines, including NCI-H187 small-cell lung cancer cells with a half-maximal inhibitory concentration (IC_50_ = 1.68 μM) and KB carcinoma cells (IC_50_ = 2.75 μM), while displaying minimal toxicity toward normal Vero cells [16]. Mechanistic investigations have revealed that 7-MH enhances tumor necrosis factor-related apoptosis-inducing ligand (TRAIL) induced apoptosis through c-Jun N-terminal kinase (JNK)-mediated upregulation of death receptor 5 (DR5) expression and caspase-3 activation in HT29 colorectal adenocarcinoma cells [17]. Furthermore, it suppresses cancer cell invasion by inhibiting matrix metalloproteinase-9 expression and modulating key signaling pathways including nuclear factor kappa B (NF-κB), signal transducer and activator of transcription 3 (STAT3), and mitogen-activated protein kinase (MAPK)/extracellular signal kinase (Erk). Molecular docking studies have identified the transforming growth factor-β-activated kinase 1 (TAK1) pathway as a primary target [18].

Current methods for extracting 7-MH from *C. harmandiana* rely on halogenated solvents, including chloroform and dichloromethane. Using these solvents results in considerable environmental and occupational health hazards, requires extended extraction times, and leads to increased energy consumption and higher operational costs [11,19,20]. In contrast, ultrasonic-assisted extraction (UAE) presents a promising green alternative. This technique employs ultrasonic waves (frequency > 20 kHz) to generate acoustic cavitation, creating microscopic bubbles that violently collapse, producing localized high temperatures and pressures. This phenomenon effectively disrupts cell walls, enhances mass transfer rates, and improves solvent penetration into the plant matrix [21]. UAE has been successfully applied to extract various alkaloid classes, including isoquinoline [22,23], protoberberine [24,25], indole [26,27], quinoline [28], quinolizidine [29], indolizidine [30], lysergic acid derivatives [31], and steroidal alkaloids [32]. The integration of UAE with response surface methodology (RSM) enables systematic optimization of extraction parameters through statistical modeling, allowing for the identification of optimal conditions while minimizing experimental trials and resource consumption.

The objectives of this study are as follows: (1) evaluate the cytotoxic efficacy of 7-MH against PANC-1 human pancreatic cancer cells under both nutrient-rich and nutrient-deprived conditions; (2) elucidate the mechanisms underlying 7-MH-induced cytotoxicity, including effects on cell morphology, colony formation capacity, and the PI3K/Akt/mTOR signaling pathway; and (3) develop and optimize an environmentally sustainable UAE method for 7-MH extraction from *C. harmandiana* root bark using RSM with central composite design (CCD).

## Materials and methods

### Plant material

*Clausena harmandiana* roots were collected from Phuwiang, Khon Kaen, Thailand. The species was identified by comparing a voucher specimen (UBU-C1) to reference specimen No. KK9807179. This reference specimen, authenticated by Prof. Dr. Pranom Chantaranothai, is deposited at Khon Kaen University. The UBU-C1 specimen is stored at the Faculty of Pharmaceutical Sciences, Ubon Ratchathani University. Following species identification, the root barks were cleaned, dried in a hot air oven at 45 °C, ground, and sieved through a 40-μm mesh sieve. The milled root bark was stored in a refrigerator (4 °C) until analysis.

### Reagents and equipment

The human pancreatic cancer cell line PANC-1 (RBRC-RCB2095) was obtained from the RIKEN BioResource Center (RBRC) cell bank (Japan). The immortalized mouse colonic epithelial cells (MCE301) were established and characterized by Professor Yoshiaki Tabuchi, Division of Molecular Genetics Research, Life Science Research Center, Organization for Promotion of Research, University of Toyama. Dulbecco’s Modified Eagle’s media (DMEM), trypsin, and a 1% antibiotics solution (1% penicillin, 1% streptomycin, and 1% amphotericin B) were purchased from Wako Pure Chemical (Osaka, Japan). The nutrient-deprived medium (NDM) was prepared in the laboratory (Toyama, Japan). A cell counting kit-8 (CCK-8), was obtained from Dojindo Laboratories (Kumamoto, Japan). Polyclonal antibodies targeting Akt, mTOR, PI3K, glyceraldehyde 3-phosphate dehydrogenase (GAPDH), phosphorylated Akt (p-AKT) at serine 473 (S473), and phosphorylated mTOR (p-mTOR) at serine 2448 (S2448) were obtained from Cell Signaling Technology (Danvers, MA, USA). A horseradish peroxidase-conjugated secondary antibody was acquired from Dako Cytomation (Glostrup, Denmark). Analytical solvents, including ethanol, methanol, hexane, ethyl acetate, and dichloromethane, were obtained from Carlo Erba (Val de Reuil, France), and high-performance liquid chromatography (HPLC) solvents, acetonitrile, formic acid, and water were purchased from Duksan Pure Chemical (Seoul, Korea). UAE was performed using a Brandson ultrasonic bath (model DT 510H, 35 kHz, 16 W). A HPLC system with an LPG-3400 pump, a WPS-3000 autosampler, a column oven, and a photodiode array detector (Dionex, Europe) was used for quality analysis of 7-MH. An octadecyl silane (ODS) HPLC column (0.46 cm × 25 cm, 5 μm) was obtained from ACE company (UK). Silica gel for column chromatography and thin-layer chromatography (TLC) materials were purchased from Merck (Darmstadt, Germany). Nuclear magnetic resonance (NMR) data were collected on a Bruker Ascend 600 MHz instrument (Scientific and Technological Research Equipment Center, Chulalongkorn University). The molecular weight (M.W.) of 7-MH was determined using a PerkinElmer CLARUS Single Quadruple 8 Gas chromatography-mass spectrometry (GC-MS) in electron ionization (EI) mode (Scientific Equipment Center, Ubon Ratchathani University).

### Ethical approval

No specific ethical approval was required for this study, as the research involved only plant material from non-endangered or protected species and *in vitro* experiments using commercially available reagents. This study did not involve human participants, human data, non-commercial human tissue, live vertebrate animals, or endangered or protected species. The human cell line used in this study was purchased from a commercial source and is considered de-identified, therefore exempt from requiring specific ethical review or informed consent.

### Isolation and characterization of 7-MH as reference standard

To obtain a pure reference compound for both anti-cancer testing and ultrasonic-assisted extraction optimization studies, traditional maceration techniques were initially employed to isolate 7-MH from *C. harmandiana* root bark. The dried root bark material (390 g) was subjected to exhaustive maceration using dichloromethane as the extraction solvent, with three sequential extractions employing 1.5 L of solvent each. The solvent was removed by using rotary evaporation, yielding 32 g of dark brown crude extract. The crude extract (30 g) was subjected to fractionation through silica gel flash column chromatography (300 g). A gradient elution system was implemented, starting with pure hexane and gradually increasing polarity through stepwise addition of ethyl acetate until reaching 100% ethyl acetate. This chromatographic separation resulted in ten distinct fractions designated Fraction 1 (F1) to Fraction 10 (F10), with the following yields: F1 – 43 mg, F2 – 52 mg, F3 – 151 mg, F4 – 104 mg, F5 – 46 mg, F6 – 127 mg, F7 – 125 mg, F8 – 430 mg, F9 – 990 mg, and F 10–730 mg. The target compound, 7-MH, was found in F8 (430 mg), which was eluted from column chromatography at 45−50% hexane in ethyl acetate. Further purification of this fraction was performed using preparative thin-layer chromatography. A 1:1 (v/v) mixture of hexane and ethyl acetate was used as the mobile phase. This purification step yielded 27 mg of pure 7-MH. Multiple analytical techniques were utilized for compound characterization. High-performance liquid chromatography (HPLC) analysis verified the compound’s purity, while structural elucidation was accomplished through comprehensive spectroscopic characterization, including proton nuclear magnetic resonance (^1^H NMR), carbon-13 nuclear magnetic resonance (^13^C NMR), and mass spectrometry (MS).

### Assessment of the anti-PANC-1 cancer cells activity of 7-MH and its mechanisms

#### Cytotoxicity assay.

Human pancreatic cancer PANC-1 cells (2 × 10^4^ cells/well) and immortalized mouse colonic epithelial cells (MCE301) (2 × 10^4^ cells/well) were cultured in DMEM, with 0.1% sodium bicarbonate, 1% antibiotics solution, and 10% fetal bovine serum (37 °C, 5% CO_2_, 24 h) in a 96-well plate [33]. PANC-1 cells were exposed to 7-MH (0–100 µM) in either NDM or DMEM for 24 h. MCE301 cells were treated with 7-MH (0–100 µM) in DMEM F-12 HAM for 24 h. After treatment, fresh DMEM containing 5% CCK-8 reagent was added. Following 3-hour incubation, cell viability was measured at 450 nm by a microplate spectrophotometer measured cells (Multiskan SkyHigh, Thermo Fisher Scientific). Cell survival (%) was calculated using GraphPad Prism [34,35].

#### Live-cell imaging and cell morphology.

PANC-1 cells (2 × 10^5^ cells/well) were placed in 35-mm dishes and incubated at 37 °C, 5% CO_2_, for 24 h. Cells were incubated in NDM containing 5 or 10 μM 7-MH and monitored in a CO_2_ incubator connected to a CytoSMART digital microscope (Lux2). A sequence of time-lapse observations was created by capturing images every 15 min for 24 h. Thereafter, cells were first fixed with 10% formaldehyde and then stained with EB and AO at 100 µg/mL each (1:1 ratio) for 15 minutes. Their images were captured under red and green fluorescence and phase-contrast modes using a digital microscope with a 20X objective (EVOS-FL). The images were analyzed using ImageJ software [34–37].

#### Wound healing assay.

An asymmetrical wound was formed in a monolayer of pancreatic cancer cells grown in a split-well insert, seeded at 1 × 10^6^ cells/well and incubated at 37 °C, 5% CO_2_, for 24 h. The cells were then exposed to 25 and 50 µM 7-MH in DMEM. Cell behavior was observed and recorded every 15 min for 24 h using a digital microscope (CytoSMART, Axion Biosystems). The wound area in each captured image was quantified using Fiji software [34,35,37].

#### Colony formation assay.

PANC-1 cells (5 × 10^3^ cells/well) were cultured in a 12-well plate using DMEM and incubated at 37 °C, 5% CO_2_, for 24 h. The cells were then exposed to 12.5, 25, or 50 µM 7-MH in DMEM for 24 h. After the medium change, the cells were allowed to grow for an additional 10 days. Following this period, the cells were stained with crystal violet solution for 15 min. The ImageJ plugin “Colony Area” was employed to quantify colony sizes [34–37].

#### Western blot analysis.

PANC-1 cells (1 × 10^6^ cells/well) were seeded in a 6-well plate and incubated at 37 °C, 5% CO_2_, for 24 h. Then, cells were treated with 7-MH in NDM (0, 5, 10, and 20 µM) or DMEM (0 and 20 µM) for 6 h. Afterward, the cells were lysed with radioimmunoprecipitation assay (RIPA) buffer, and proteins were extracted. The lysates were subjected to sodium dodecyl sulfate polyacrylamide gel electrophoresis (SDS-PAGE) and transferred to polyvinylidene difluoride (PVDF) membranes. The membranes were blocked with 5% skim milk in Tris buffered saline with Tween 20 (TBST), probed with primary antibodies, and washed with TBST. Next, secondary antibodies were added. Bands were visualized using a chemiluminescence solution (Bio-Rad, USA), and their densities were measured using Fiji software [34–36].

### HPLC method for 7-MH analysis

HPLC was used to identify and quantify 7-MH. The stationary phase was an ODS type column. The mobile phase was a combination of 0.1% formic acid (A) and acetonitrile (B). A gradient elution was performed with changing solvent composition as follows: 0–5 min: 95% A, 5–10 min: 85% A, 10–15 min: 78% A, 15–20 min: 75% A, 20–40 min: 30% A, and 40–50 min: 95% A. The solvent mixture flowed at a rate of 0.8 mL/min. The detection wavelength was 270 nm. The validity of the HPLC analysis method was assessed by specificity, linearity, precision, accuracy, detection limit, and quantification limit, in accordance with International Conference on Harmonization (ICH) Q2 (R1) guidelines [38].

### Optimization of UAE for 7-MH extraction

A safer and more efficient solvent was tested for 7-MH extraction, aiming to improve the ecological extraction method. The extraction abilities of ethanol and dichloromethane were compared using two test sets (n = 3, each). Each sample tube contained 500 mg of root bark powder and 10 mL of solvent (dichloromethane for set A and absolute ethanol for set B). Both sample sets were sonicated in a Brandson ultrasonic bath (35 kHz, 16 W) at 70°C for 20 min, followed by the quantification of 7-MH content using HPLC. Preliminary analysis revealed that using dichloromethane (0.60 ± 0.04% w/w) produced more 7-MH yield than ethanol (0.52 ± 0.17% w/w), but this difference was not statistically significant (*p* > 0.05, independent t-test). Based on the preliminary test results, ethanol was chosen as the preferred extraction solvent. Conditions for 7-MH extraction were experimentally optimized using UAE and RSM with a CCD experiment. Extractions were performed with varying levels of three variables: $X_{\text{1}}$= temperature of sonication (°C), $X_{\text{2}}$= time of sonication (min), and $X_{\text{3}}$ = plant/solvent ratio (P/S ratio, g/10 mL). The variables were coded with five levels: + 1, −1, 0, + 1.68, −1.68 (*α* and –*α* respectively) (Table 1). After sonication, the supernatant was filtered, and 7-MH content was determined using HPLC. The data were analyzed for their determination coefficient (R²), adjusted determination coefficient (adjusted R²), and coefficient of variation (CV, %) and subjected to analysis of variance (ANOVA). These analyses were used to establish a second-degree polynomial regression model for predicting the 7-MH yield.

**Table 1:** Coded and actual levels of three independent variables.

Variables	Coded levels of variables				
	–1.68	–1	0	1	1.68
Temperature of sonication ($X_{\text{1}}$)	23	30	40	50	57
Time of sonication ($X_{\text{2}}$)	0	15	38	60	75
Plant/solvent (P/S) ratio (g/10 mL) ($X_{\text{3}}$)	0.10	0.20	0.35	0.50	0.60

### Statistical analysis

Extraction conditions and their results were optimized and analyzed using Version 23.1 of Stat-Ease Design-Expert software. The preliminary results of the extraction solvents analysis were analyzed using independent samples t-test (two-tail), with **p* *< 0.05 considered significant (Microsoft Excel 2019). Experimental data were collected in triplicate and presented as mean ± SD. Cell survival and wound healing data were processed and calculated using GraphPad Prism V. 10.1.

## Results

### Isolation and characterization of 7-MH as reference standard

7-MH was purified from *C. harmandiana* extract. Fractionation of 30 g of crude extract via silica gel flash column chromatography using a hexane-ethyl acetate gradient system produced 10 fractions (F1–F10) with yields ranging from 43 mg to 990 mg. F8 (430 mg) was identified as containing the target compound based on its HPLC-UV spectrum and was further purified by preparative thin-layer chromatography using hexane-ethyl acetate as the mobile phase. This purification yielded 27 mg of 7-MH as pale yellow crystals. The high purity of the isolated compound was determined by HPLC analysis. Its structure was confirmed by MS, ^1^H NMR and ^13^C NMR data (S1 Fig and S1 Table), with all spectroscopic data in good agreement with values presented in the literature [16]. This purified 7-MH served as the reference standard for anti- cancer and ultrasonic-assisted extraction optimization studies.

### Anti-cancer activity of 7-MH against PANC-1 pancreatic cancer cells and the underlying mechanism

#### Cytotoxic effects of 7-MH.

**Fig 1:**
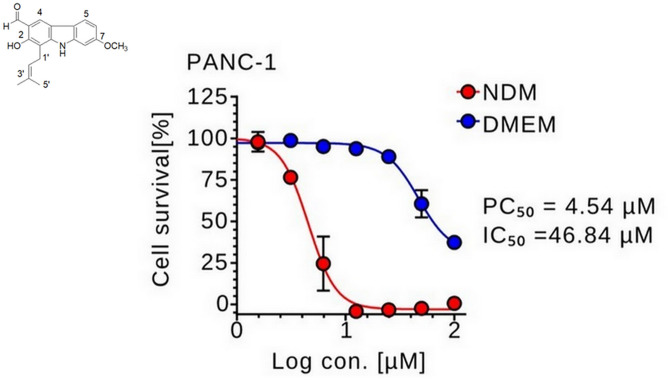
7.MH Exhibits Anti-PANC-1 Cell Activity in NDM and DMEM.

**Fig 2:**
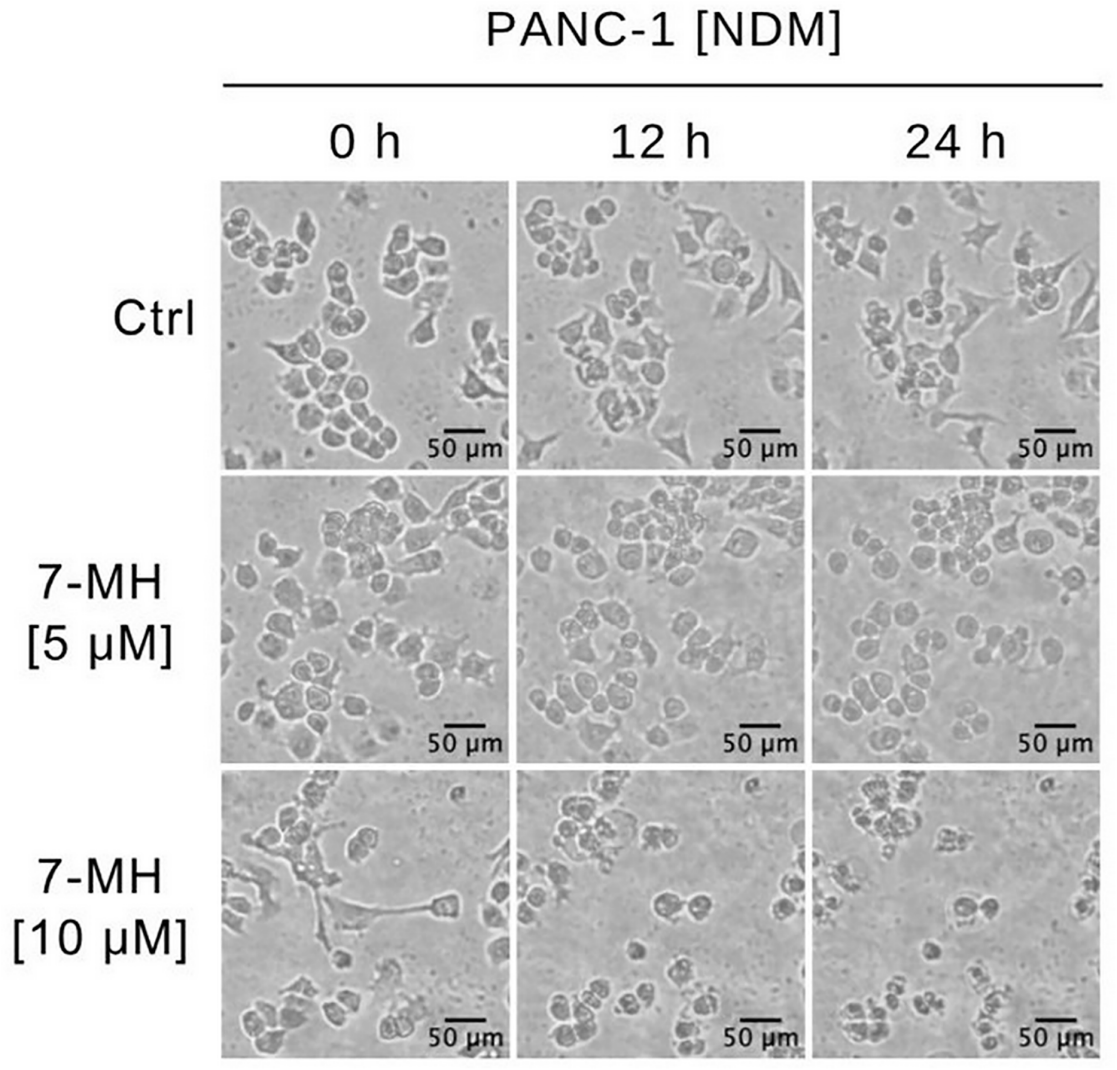
Live-Cell Imaging of the Effect of 7-MH (0, 5, or 10 µM) on PANC-1 Cells in NDM Over Time.

**Fig 3:**
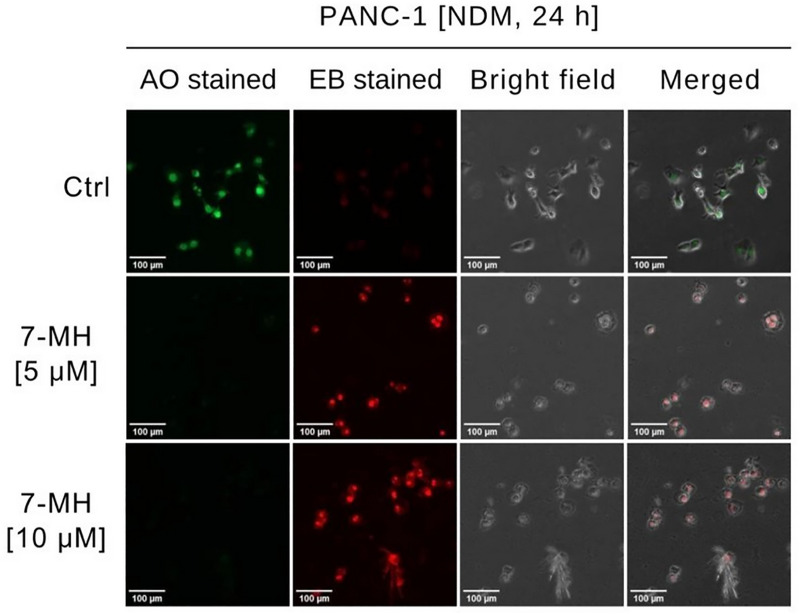
Morphological Changes in PANC-1 Cells Treated with 7-MH (5 and 10 µM) in NDM for 24 h compared with untreated cells (Ctrl).

PANC-1 cells were treated with various concentrations of 7-MH in 96-well plates for 24 h. As shown in Fig 1, 7-MH showed differential cytotoxicity under different nutritional conditions. The PC_50_ (preferential cytotoxicity at 50%) value represents the concentration at which 7-MH selectively eliminated 50% of PANC-1 cells cultured in nutrient deprived medium (NDM), while the IC_50_ value indicates the concentration required to induce 50% cytotoxicity when cells were cultured in nutrient-rich medium (DMEM). Under nutrient-deprived condition, 7-MH exhibited a PC_50_ value of 4.54 µM in NDM, whereas significant cytotoxicity in nutrient-rich DMEM was observed at higher concentrations, with an IC_50_ value of 46.84 µM. To identify anti-austerity agents, a selective index is used. The index is calculated by dividing the IC_50_ of a compound in DMEM by its PC_50_ in NDM. A high selective index suggests the compound is more toxic to PANC-1 cells under nutrient-deprived conditions. In this study, 7-MH had a selective index of 10.32, which showed its effectiveness as an anti-austerity agent. 7-MH’s toxicity was also tested on the immortalized mouse colonic epithelial cell line (MCE301). The results (Supplementary S2 Fig) demonstrated that 7-MH showed minimal cytotoxic effects against normal cells (IC_50_ = 83.4 µM), supporting the toxicity of this compound against PANC-1 cancer cells. To further evaluate anticancer activity of 7-MH, concentrations were selected based on an anti-austerity strategy, targeting pancreatic cancer cells under both nutrient-deprived and nutrient-rich conditions. In NDM, concentrations of 5 and 10 µM, near the PC₅₀ value (4.54 µM), were selected to evaluate cytotoxicity under conditions mimicking the hypovascular tumor microenvironment. In nutrient-rich DMEM, higher concentrations approaching the IC₅₀ value (46.84 µM) were used for wound healing and colony formation assays to investigate effects on cancer cell migration and proliferation, reflecting the nutrient-rich environments of metastatic sites such as the liver, lungs, and duodenum. This approach ensures alignment with the anti-austerity strategy and the biological context of pancreatic cancer metastasis. To investigate the apoptotic morphological changes and cell death induced by 7-MH in PANC-1 cells under nutrient starvation, real time live-cell imaging was conducted. The PANC-1 cells were treated with 0 (untreated cells), 5, or 10 µM of 7-MH and monitored over a 24 h period. The untreated cells maintained normal morphology and viability throughout the experiment (Fig 2). In contrast, cells treated with 5 µM of 7-MH exhibited rounded morphology, while those treated with 10 µM of 7-MH showed pronounced apoptotic features, including cell shrinkage and plasma membrane blebbing, which appeared within 12 h and culminated in complete cell death by 24 h. These dynamic changes are illustrated in S1 File. To determine the effects of 7-MH on the morphology and survival of PANC-1 cells, ethidium bromide/acridine orange (EB/AO) double staining was employed. This technique differentiates live cells from dead cells based on membrane integrity: acridine orange stains the cell nuclei green, while ethidium bromide only enters cells with damaged membranes, staining their nuclei red. Thus, green fluorescence indicates viable cells, and red fluorescence indicates cytotoxicity. After 24 h, the untreated cells maintained normal morphology with no EB staining. Meanwhile, the cells exposed to 5 or 10 µM 7-MH displayed abnormal morphology and red fluorescence due to EB uptake, indicating loss of membrane integrity and cell death (Fig 3).

#### Inhibition of PANC-1 cell migration and colony formation by 7-MH.

**Fig 4:**
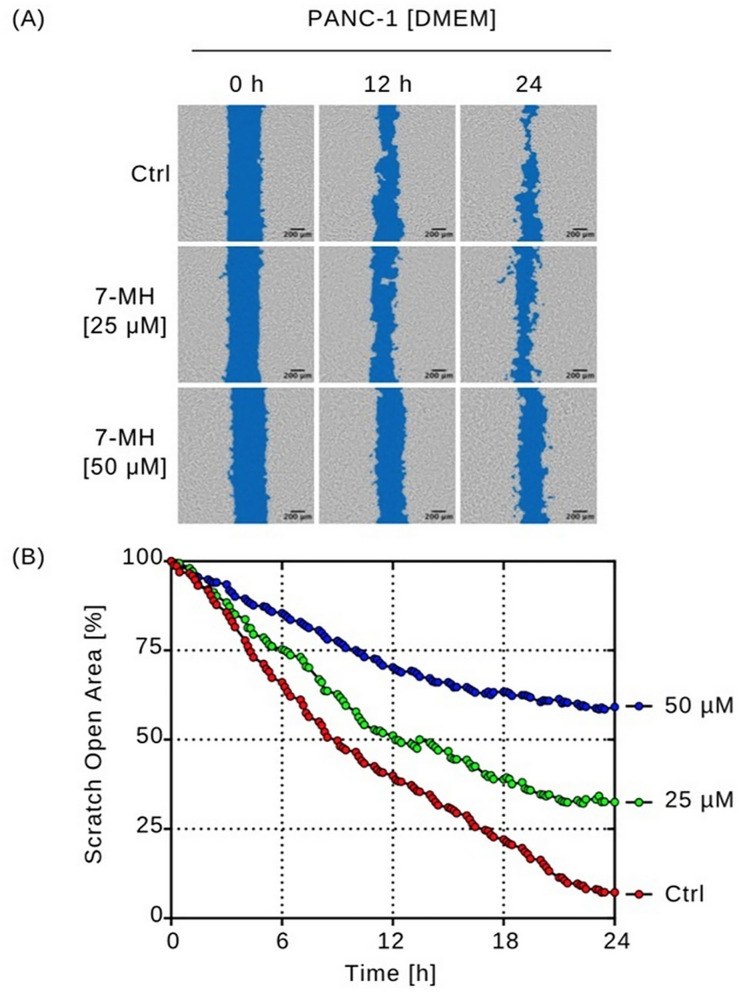
Effect of 7-MH on pancreatic cancer cell migration. **(A)** The wound healing effect at 0, 12, and 24 h was assessed using real-time imaging. **(B)** Cancer cells migration was quantified by calculating the open wound area at 15-min intervals for 24 **h.**

**Fig 5:**
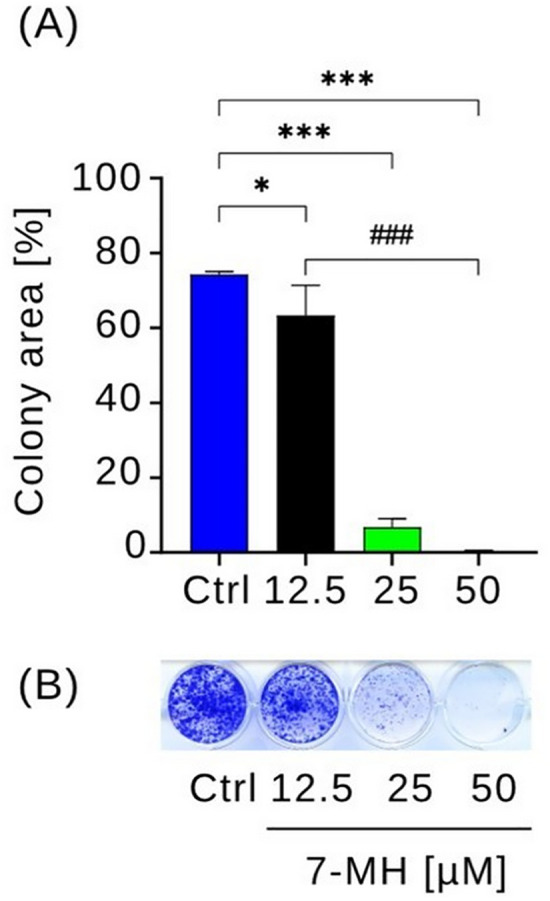
Effect of 7-MH on PANC-1 cell colony formation. PANC-1 cells were treated with 7-MH at concentrations of 12.5, 25, and 50 μM and compared to untreated cells (Ctrl). **(A)** Mean colony areas, data are presented as a percentage ± SD (n = 3). **(B)** Representative images of colonies. Statistical significance: *p < 0.01 for 12.5 μM vs. Ctrl; ***p < 0.001 for 25 and 50 μM vs. Ctrl; ^*###*^*p* < 0.001 between treatment groups.

Pancreatic cancer cells often migrate from the nutrient-deficient tumor microenvironment to nutrient-rich organs such as the liver and stomach [39]. To evaluate the potential inhibitory effect of 7-MH on PANC-1 cells migration, a wound healing assay was performed in nutrient rich DMEM. Cells were seeded into split-well culture inserts to create defined cell-free gaps, then treated with 7-MH (25 or 50 µM) or left untreated as controls. The test concentrations were selected based on 7-MH IC_50_ value determined in nutrient-rich conditions. Migration was monitored over 24 h in a CO_2_ incubator, a duration that was sufficient to observe phenotypic changes in cell migration and proliferation [34,35]. Images of the untreated and treatment groups were captured every 15 min to monitor wound healing (Fig 4 and S2 File). After 24 h, wounds in the untreated group were nearly completely closed, whereas groups treated with 25 and 50 µM of 7-MH showed remaining wound areas of 32% and 59%, respectively. These results indicate that 7-MH inhibited PANC-1 cell migration in a dose dependent manner. For further investigation, the effect of 7-MH on colony formation was evaluated (Fig 5). 7-MH significantly inhibited cell colony formation. While untreated cells covered approximately 75% of the plate area, treatment with 12.5 µM concentration of 7-MH reduced colony formation to 63% (**p* *< 0.01). A stronger inhibitory effect was observed at 25 µM, where colony coverage dropped to 6.7% (*p* < 0.001). In addition, a 50 µM concentration of this compound completely inhibited the formation of any cell colonies (**p* *< 0.001).

#### Effect of 7-MH on PI3K/Akt/mTOR signaling pathway protein expression.

**Fig 6:**
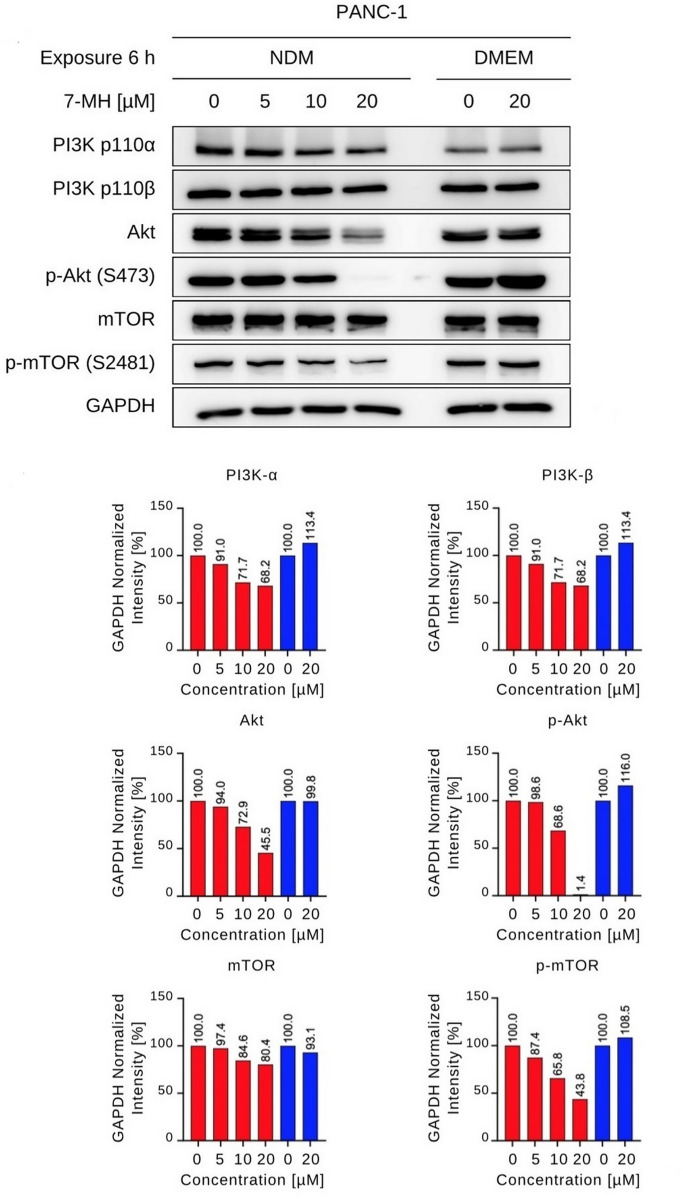
Effect of 7-MH on PI3K/Akt/mTOR Signaling Pathway. Band intensities were normalized to GAPDH intensity.

The PI3K/Akt/mTOR signaling pathway is frequently activated in pancreatic cancer, where it plays a critical role in promoting cellular tolerance to nutrition deprivation within the tumor microenvironment [40]. Therefore, to investigate whether 7-MH modulates key proteins in this pathway, western blot analysis was performed. PANC-1 cells were treated with 7-MH at concentrations of 5, 10, and 20 µM under NDM, or with 20 µM under DMEM for 6 h. The 6 h timepoint was chosen to capture early signaling events within the PI3K/Akt/mTOR cascade, as reported in previous studies [34,35]. This duration allows detection of protein expression changes while avoiding confounding effects from secondary cellular responses or cell death that could obscure mechanistic insights. As shown in Fig 6, 7-MH treatment in DMEM did not alter Akt, p-Akt, or p-mTOR expression. In contrast, under NDM conditions, 20 µM of 7-MH downregulated Akt by 45% and p-mTOR by 43% and completely inhibited p-Akt. Original Western blot images for protein expression are provided in the Supplementary information (S1 Data).

### HPLC method for 7-MH analysis

**Fig 7:**
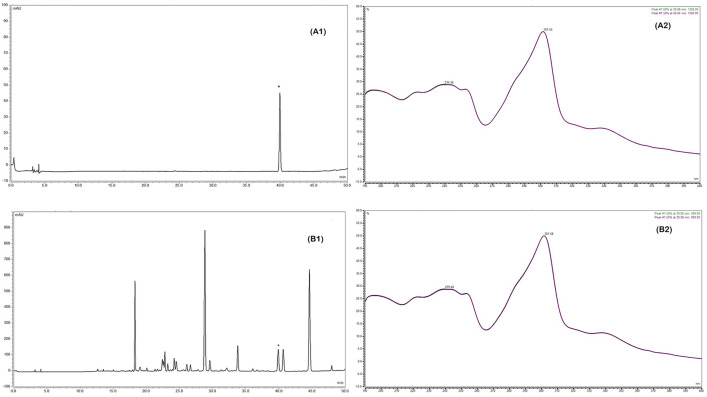
HPLC Chromatograms and UV spectra of Reference Standard 7-MH and 7-MH from Ethanol Extract. (A1) HPLC chromatogram and (A2) UV spectrum of reference standard 7-MH. (B1) HPLC chromatogram of the ethanol extract containing 7-MH and (B2) UV spectrum of the identified 7-MH peak within the ethanol extract.

The HPLC analysis method for 7-MH was validated according to ICH Q2 (R1) guidelines. Isolated 7-MH was used as a reference standard. This compound displayed a single peak in the HPLC chromatogram at a retention time of 39.8 min (Fig 7). Standard curves were constructed within the linear range of 5–100 µg/mL for the compound, demonstrating a strong coefficient value (R^2^ > 0.995) as shown in Table 2. The method exhibited high sensitivity for the analyte, with a detection limit of 0.20 µg/mL and a quantitation limit of 0.61 µg/mL. Intra- and inter-day precision values were 0.76% RSD and 1.19% RSD, respectively, indicating both good repeatability and intermediate precision. Accuracy was confirmed by spiking the samples with three different concentrations of 7-MH. The recoveries of the spiked compounds ranged from 93% to 98% with % RSD values less than 3%, demonstrating good accuracy. These results indicate that the method is suitable for determining 7-MH content in the analyzed extracts.

**Table 2:** Parameters of HPLC Method Validation for 7-MH Analysis.

Linearity		
**Standard curve** (n = 3)	**Regression equation**	**r** ^**2**^
1	y = 0.3336x + 0.3707	0.9998
2	y = 0.3386x + 0.4111	0.9996
3	y = 0.3377x + 0.3977	0.9998
**Precision**		
	**Mean ± SD (µg/mL)**	**RSD (%)**
Intra-day (n = 6)	0.94 ± 0.01	0.76
Inter-day (n = 15)	0.93 ± 0.01	1.19
**Accuracy**		
**Spiked concentration (µg/mL)**	**Recovery (%)**	**RSD (%)**
2.5	93.84 ± 1.51	1.61
5	98.22 ± 1.73	1.76
10	96.60 ± 2.46	2.54

### Optimization of UAE of 7-MH

#### UAE conditions for 7-MH extraction.

RSM with a CCD was applied to optimize UAE conditions for 7-MH extraction from *C*. *harmandiana* root bark. The role of three parameters, including temperature of sonication ($X_{\text{1}}$), time of sonication ($X_{\text{2}}$), and plant/solvent ratio ($X_{\text{3}}$), on the response (y) 7-MH yield was investigated. In total, 17 runs were tested according to the CCD plan. In these experiments, 7-MH yields ranged from 0.48% to 1.28% w/w (Table 3).

**Table 3:** Comparison of actual and predicted 7-MH yields using CCD experiments.

Experiment	Coded levels of variables			Predicted yield (% w/w)	Actual yield (% w/w)
	$X_{1}$	$X_{2}$	${\;X}_{3}$		
**1**	–1	–1	–1	1.05	1.03 ± 0.07
**2**	1	–1	–1	0.72	0.72 ± 0.01
**3**	–1	1	–1	1.14	1.15 ± 0.01
**4**	1	1	–1	0.93	0.96 ± 0.06
**5**	–1	–1	1	0.46	0.48 ± 0.01
**6**	1	–1	1	0.62	0.58 ± 0.01
**7**	–1	1	1	0.93	0.86 ± 0.03
**8**	1	1	1	1.22	1.20 ± 0.00
**9**	–1.68	0.00	0	1.16	1.12 ± 0.02
**10**	1.68	0.00	0	1.11	1.15 ± 0.14
**11**	0	–1.68	0	0.68	0.69 ± 0.05
**12**	0	1.68	0	1.27	1.28 ± 0.01
**13**	–1.68	0	–1.68	1.04	1.06 ± 0.02
**14**	1.68	0	1.68	0.75	0.76 ± 0.01
**15**	0	0	0	1.19	1.20 ± 0.26
**16**	0	0	0	1.19	1.16 ± 0.01
**17**	0	0	0	1.19	1.22 ± 0.13

#### Experimental data analysis.

Analysis of the optimization data revealed that a quadratic model was adequate for establishing the regression model and describing the interaction between the variables and extraction yield. The quadratic regression model is represented by Equation (1).


$$y=\text{1}.\text{1}\text{9}-\text{0}.\text{0}\text{1}\text{3}X_{\text{1}}+\text{0}.\text{1}\text{7}\text{4}X_{\text{2}}-\text{0}.\text{0}\text{7}\text{5}X_{\text{3}}+\text{0}.\text{0}\text{2}\text{7}X_{\text{1}\;}X_{\text{2}}+\text{0}.\text{1}\text{2}\text{3}X_{\text{1}}X_{\text{3}\;}+\text{0}.\text{0}\text{9}\text{8}X_{\text{2}\;}X_{\text{3}}-\text{0}.\text{0}\text{1}\text{8}X_{\text{1}}^{\text{2}}-\text{0}.\text{0}\text{7}\text{4}X_{\text{2}}^{\text{2}}-\text{0}.\text{2}\text{0}\text{8}X_{\text{3}}^{\text{2}}$$ (1)

**Fig 8:**
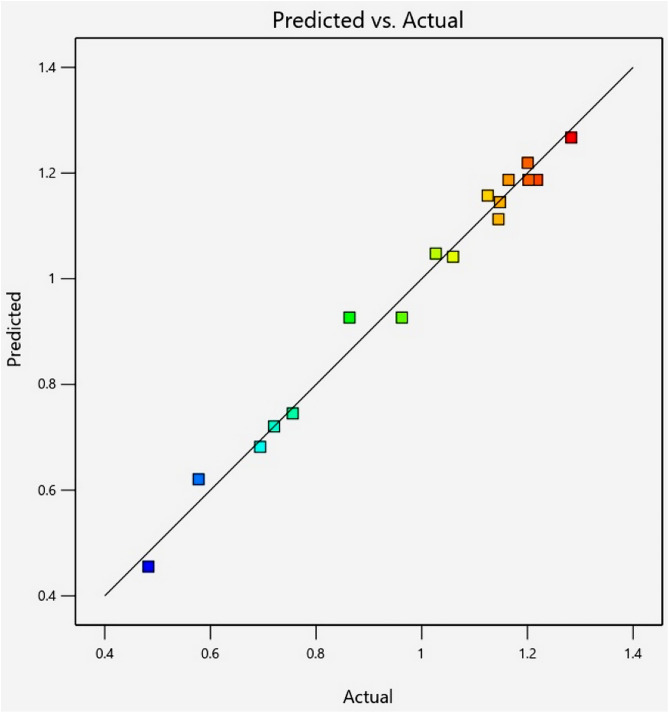
Correlation plot of the actual and predicted yields of 7-MH using UAE.

ANOVA was used for statistical analysis. The model demonstrated a good fit, as evidenced by its significance (**p <* *0.05), along with a non-significant lack of fit (**p >* *0.05). The determination coefficient of the model (R^2^) indicated a positive linear correlation between the actual and predicted data, as shown in Fig 8. The positive adjusted R^2^ and the low CV confirmed the highly significant fit of the model. The parameters $X_{\text{2}}$, $X_{\text{3}}$, $X_{\text{1}}X_{\text{3}}$, $X_{\text{2}\;}X_{\text{3}}$, $X_{\text{2}}^{\text{2}}$, and $X_{\text{3}}^{\text{2}}$were statistically significant (*p <* 0.05), demonstrating the impact of these factors on the extraction yield (Table 4).

**Table 4:** ANOVA for 7-MH yield.

Source	Coefficient	Sum of Squares	Degrees of Freedom	Mean Square	F-value	*P*-value
Model	1.19	0.9742	9	0.1082	56.79	<0.0001
$X_{\text{1}}$	–0.013	0.0022	1	0.0022	1.16	0.3176
$X_{\text{2}}$	0.174	0.3177	1	0.3177	166.69	<0.0001
$X_{\text{3}}$	–0.075	0.0475	1	0.0475	24.9	0.0016
$X_{\text{1}\;}X_{\text{2}}$	0.027	0.0043	1	0.0043	2.26	0.1766
$X_{\text{1}}X_{\text{3}\;}$	0.123	0.1007	1	0.1007	52.84	0.0002
$X_{\text{2}\;}X_{\text{3}}$	0.098	0.0560	1	0.056	29.39	0.001
$X_{\text{1}}^{\text{2}}$	–0.018	0.0033	1	0.0033	1.75	0.2271
$X_{\text{2}}^{\text{2}}$	–0.074	0.0552	1	0.0552	28.98	0.001
$X_{\text{3}}^{\text{2}}$	–0.208	0.1915	1	0.1915	100.45	<0.0001
Residual		0.0133	7	0.0019		
Lack of fit		0.0069	4	0.0017	0.8022	0.5962
Pure error		0.0064	3	0.0021		
Corrected total sum of squares		0.9875	16			
R^*2*^		0.9865				
Adjusted R^*2*^		0.9691				
CV (%)		4.46				

#### Response surface analysis.

**Fig 9:**
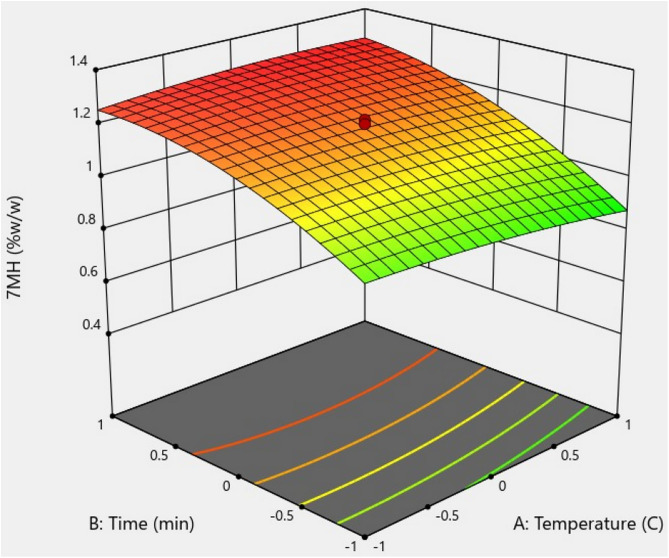
Response surface plot showing the effect of the relationship between temperature and time of sonication on 7-MH yield.

**Fig 10:**
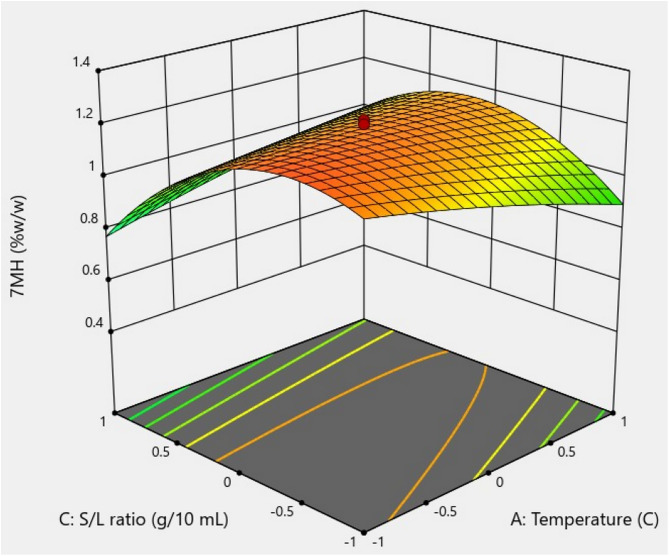
Response surface plot showing the effect of the interaction of temperature of sonication and P/S Ratio on 7-MH yield.

**Fig 11:**
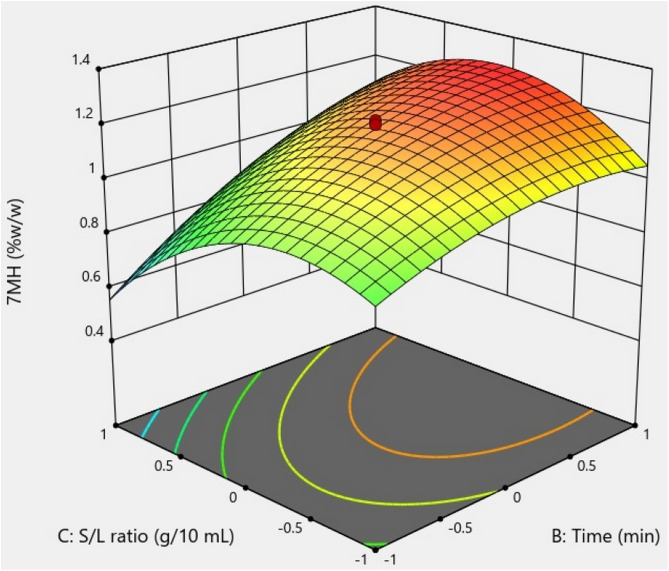
Response surface plot showing the effect of the interaction of time of sonication and P/S ratio on 7-MH yield.

Three-dimensional surface plots revealed the factors that influence the 7-MH extraction yield. Fig 9 presents the relationship between temperature and time interaction during sonication, which did not significantly improve the yield of 7-MH (*p >* 0.05). Fig 10 shows the temperature and P/S ratio interaction, which did affect the 7-MH yield. The 7-MH yield was high in the temperature range of 30–50 °C and at P/S ratios of 0.23–0.29 g/10 mL. Outside these ranges, 7-MH content tended to decrease. Fig 11 demonstrates the effect of time and P/S ratio on 7-MH yield. The extraction yield of 7-MH was high within the time range of 50–60 min and P/S ratio range of 0.28–0.43 g/10 mL.

#### Model validation.

The optimal extraction conditions to maximize 7-MH yield were determined using regression equation (1). Under the optimal conditions, ultrasonic frequency: 35 kHz, temperature: 50 °C, time: 60 min, and plant/solvent ratio: 0.40 g/10 mL, the 7-MH content in the test set (n = 3) was 1.26 ± 0.02% w/w. This value was close to the predicted value of 1.31% w/w, obtained from Equation 1. The percentage error was 3.55%. Thus, the validation data demonstrated the usability of this regression model for accurate and reliable UAE process optimization.

## Discussion

The aggressive behavior of pancreatic cancer is one factor that results in short survival times for patients after diagnosis. This behavior, including a high mutation rate during progression, resistance to standard therapies like chemotherapy and radiation, austerity, and early metastasis, contributes to its severity [41]. Plant-derived compounds serve as potential sources of anti-pancreatic cancer agents [4,34,35,42–45]. In this study, 7-MH exhibited selective cytotoxicity against PANC-1 cells, showing a preferential cytotoxicity (PC_50_) of 4.54 µM under nutrient-deprived conditions (NDM). This was significant lower concentration than the IC_50_ of 46.84 µM required to induce cytotoxicity in nutrient-rich medium (DMEM). Furthermore, 7-MH demonstrated minimal toxicity to the normal MCE301 cells (IC_50 _= 83.4 µM), suggesting 7-MH may act as a selective therapeutic agent against PANC-1 pancreatic cancer, particularly under conditions that mimic the tumor microenvironment. Real-time imaging showed that 7-MH dose-dependently induced apoptosis in PANC-1 cells within 24 h under NDM. which was characterized by membrane blebbing, cell shrinkage, and the loss of membrane integrity. These results agree with a previous report on 7-MH-induced apoptosis in HT-29 colorectal adenocarcinoma cells [17]. In addition, 7-MH at 25 and 50 µM significantly impaired cancer cell migration and inhibited colony formation. The complete suppression of colony formation at 50 µM suggests potent inhibition of clonogenic potential, a hallmark of cancer stem-like cells linked to self-renewal and tumor initiation. Similarly, inhibition of wound healing at this concentration indicates reduced migratory capacity, another characteristic of stem-like cells associated with metastasis. Conducted in nutrient-rich DMEM, these assays reflect environments like metastatic sites (e.g., liver or lungs) where cancer stem-like cells thrive, making them relevant for evaluating 7-MH’s effects on aggressive cancer cell behaviors. However, direct evidence for targeting cancer stem-like cells requires further investigation, such as sphere formation assays or analysis of stemness markers (e.g., CD44, CD133) [46,47].

Moreover, the anti-PANC-1 effects of 7-MH are mechanistically involved in the suppression of the PI3K/Akt/mTOR signaling pathway. which is critical for cell survival, proliferation, growth, metabolism, and motility. The phosphorylation of these proteins regulates diverse cellular functions, such as transcription and translation. The recurrent hyperactivation of this pathway in cancer establishes it as an attractive therapeutic target. This is further supported by ongoing clinical trials of several PI3K, Akt, and mTOR inhibitors for pancreatic cancer treatment [48–50]. Under nutrient-deprived media conditions, 20 µM of 7-MH downregulated Akt by 45% and p-mTOR by 43% while completely inhibiting p-Akt. In contrast, this effect was not observed when cells were cultured in nutrient-rich media. Nutrient deprivation induces metabolic stress in pancreatic cancer cells, increasing their dependence on survival pathways like PI3K/Akt/mTOR [40]. 7-MH selectively inhibits this pathway under nutrient-deprived conditions, as evidenced by the low PC₅₀ value (4.54 µM) in NDM compared to the higher IC₅₀ value (46.84 µM) in DMEM, explaining its enhanced cytotoxicity. The high selective index 10.32 suggests that metabolic stress sensitizes the cells to 7-MH by disrupting critical survival signaling. Additionally, adaptive mechanisms, such as autophagy or mitochondrial metabolism, may contribute to cancer cell survival under nutrient stress, warranting further investigation. These findings align with previous research on kigamicin D, an agent derived from actinomycetes that targets the PI3K/AKT signaling pathway in PANC-1 cancer cells under nutrient deprived conditions [51]. The experimental results therefore position 7-MH as a promising anti-austerity agent for future research into pancreatic cancer treatments. However, there are limitations of this study, including the 6-hour PI3K/Akt/mTOR analysis, which may miss long-term effects, and the lack of direct assays (e.g., sphere formation, CD44/CD133 analysis) to confirm cancer stem-like cell inhibition [52]. *In vitro* models are also unable to fully replicate the complexity of the tumor microenvironment [53,54]. Therefore, *in vivo* studies are essential to validate efficacy, pharmacokinetics, and safety of 7-MH. Future research addressing these constraints is critical for its potential clinical translation.

7-MH was traditionally extracted from *C. harmandiana* root bark using the tedious maceration or Soxhlet method with halogenated solvents. To develop a more efficient and environmentally sustainable method for 7-MH extraction, this study employed UAE in conjunction with RSM and a CCD to optimize conditions for extraction from *C. harmandiana* root bark. The results were used to create a prediction model that accurately predicted the extraction yield, as indicated by its significance (**p* *< 0.05), a lack of fit with *p* > 0.05, R² of 0.9865, adjusted R² of 0.9691, and a CV of 4.46%. The factors affecting 7-MH extraction yield included time of sonication ($X_{\text{2}}$), P/S ratio ($X_{\text{3}}$), temperature of sonication and P/S ratio interaction ($X_{\text{1}}X_{\text{3}}$), and time of sonication and P/S ratio interaction ($X_{\text{2}}X_{\text{3}}$). Increasing the P/S ratio from 0.2 to 0.4 g/10 mL resulted in a higher 7-MH extraction yield (*p <* 0.05). Conversely, a P/S ratio > 0.5 g/10 mL did not improve the extraction yield. This result suggests that an appropriate plant sample and extraction solvent ratio is crucial for efficient extraction, as previously reported in the literature [25,55,56]. Furthermore, 7-MH yield increased with time of sonication. A prolonged sonication time can enhance solvent penetration to dissolve the target compound from the plant cells. However, an excessively long sonication time results in decreased yield due to saturation of compound solubility in both the plant cells and solvent. This finding is consistent with findings on *Mitragyna* leaf extraction by Zakaria et al. [57], who reported that a higher leaf-to-solvent ratio required longer extraction times for a higher alkaloid yield. Theoretically, an increase in sonication temperature stimulates cavitation and results in increased compound yield. In the present study, temperature did not significantly affect the 7-MH yield, which can be attributed to solvent volatility at high temperatures [58]. This result aligns with findings previously reported by Zhong et al. [55] on the extraction of alkaloids from *Macleaya cordata* fruits. To validate the regression model, the predicted sonication condition for maximizing 7-MH yield was used (temperature: 50 °C, time: 60 min, and P/S ratio: 0.40 g/10 mL). The extracted 7-MH yield was 1.26 ± 0.01% w/w, with a percentage error of less than 5%. Therefore, these findings confirm the regression model is suitable for predicting 7-MH yield.

## Conclusion

7-Methoxyheptaphylline from *Clausena harmandiana* demonstrates significant anti-cancer activity against PANC-1 pancreatic cancer cells. The compound exhibited selective cytotoxicity and induced apoptosis in PANC-1 cells. 7-MH effectively impaired cancer cell proliferation by inhibiting wound closure and colony formation and mechanistically suppressed the PI3K/Akt/mTOR pathway. To support the sustainable use of this compound, an efficient and environmentally friendly ultrasonic-assisted extraction method was developed using ethanol, avoiding the use of halogenated solvents. This research not only establishes 7-MH as a potential natural compound for future pancreatic cancer studies but also provides a model for the sustainable application of green chemistry in natural product extraction.

## Supporting information

S1 Table ^1^H and ^13^C NMR chemical shifts of isolated 7-MH in CDCl_3_ compared with the reference. (DOCX)

S1 Fig Mass spectrum of 7-MH. (TIF)

S2 Fig Cytotoxicity activity of 7-MH on MCE301 colonic epithelial cell line. (TIF)

S1 File Effects of 7-MH on pancreatic cancer cell morphology and cell death. (MP4)

S2 File Inhibition of pancreatic cancer cell migration and colony formation by 7-MH. (MP4)

S1 Data Raw images of Western blot experiment. (TIF)
