# Supplementary material for: Anti-cancer activity of 7-methoxyheptaphylline from Clausena harmandiana against PANC-1 pancreatic cancer cells and its sustainable extraction method
Source: PLoS One. 2025 Oct 16;20(10):e0334901. doi: 10.1371/journal.pone.0334901 (PMC12530583; doi:10.1371/journal.pone.0334901)
Supplement: S1 Table — (DOCX) [file pone.0334901.s001.docx]

**S1 Table ^1^H and ^13^ C NMR chemical shifts of isolated 7-MH in CDCl_3_ compared with the reference**

| **Position** | **Isolated 7-MH** | | **Reference [16]** | |
| --- | --- | --- | --- | --- |
|  | **δ_H_ (ppm)** | **δ_C_ (ppm)** | **δ_H_ (ppm)** | **δ_C_ (ppm)** |
| 1 |  | 109.0 |  | 109.0 |
| 1a |  | 145.2 |  | 145.2 |
| 2 |  | 157.3 |  | 157.3 |
| 3 |  | 115.3 |  | 115.3 |
| 4 | 7.89 (1H, *s*) | 124.0 | 7.88 (1H, *s*) | 124.0 |
| 4a |  | 117.5 |  | 117.5 |
| 5 | 7.82 (1H, *d*, *J*=8.5 Hz) | 120.5 | 7.81 (1H, *d*, *J*=8.5 Hz) | 120.5 |
| 5a |  | 117.2 |  | 117.2 |
| 6 | 6.86 (1H, *dd*, *J*=8.5, 2.2 Hz) | 108.9 | 6.87 (1H, *dd*, *J*=8.5, 1.8 Hz) | 108.9 |
| 7 |  | 158.9 |  | 159.0 |
| 8 | 6.89 (1H, *d*, *J*=2.2 Hz) | 95.6 | 6.89 (1H, *d*, *J*=1.6 Hz) | 95.6 |
| 8a |  | 141.4 |  | 141.5 |
| 1*ꞌ* | 3.61 (2H, *d*, *J*=6.9 Hz) | 22.78 | 3.60 (2H, *d*, *J*=6.8 Hz) | 22.8 |
| 2*ꞌ* | 5.32 (1H, *t*, *J*=6.9 Hz) | 121.3 | 5.30 (1H, *t*, *J*=6.8 Hz) | 121.3 |
| 3*ꞌ* |  | 134.1 |  | 134.1 |
| 4*ꞌ* | 1.76 (3H, *s*) | 25.7 | 1.76 (3H, *s*) | 25.7 |
| 5*ꞌ* | 1.77 (3H, *s*) | 18.1 | 1.88 (3H, s*)* | 18.1 |
| 2-OH | 11.63 (1H, *s*) |  | 11.62 (1H, s) |  |
| 3-CHO | 9.87 (1H, *s*) | 195.4 | 9.86 (1H, *s*) | 195.3 |
| NH | 8.15 (1H, *br*) |  | 8.16 (1H, *br*) |  |
| 7-OCH_3_ | 3.89 (3H, *s*) | 55.7 | 3.90 (3H, s) | 55.7 |
