# Supplementary figures and images for: Anti-cancer activity of 7-methoxyheptaphylline from Clausena harmandiana against PANC-1 pancreatic cancer cells and its sustainable extraction method
Source: PLoS One. 2025 Oct 16;20(10):e0334901. doi: 10.1371/journal.pone.0334901 (PMC12530583; doi:10.1371/journal.pone.0334901)

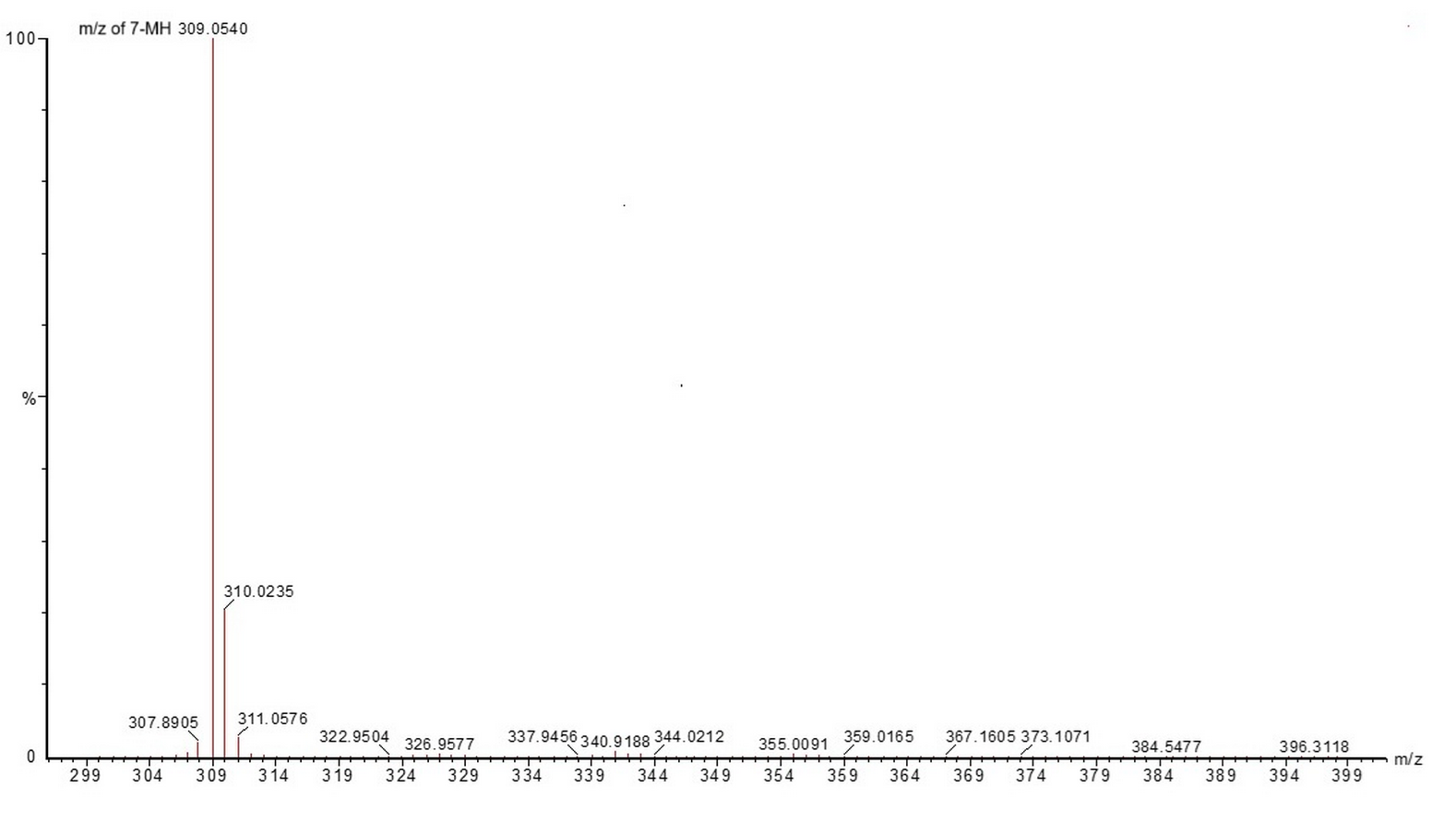

Supplement: S1 Fig — (TIF) [file pone.0334901.s002.tif]

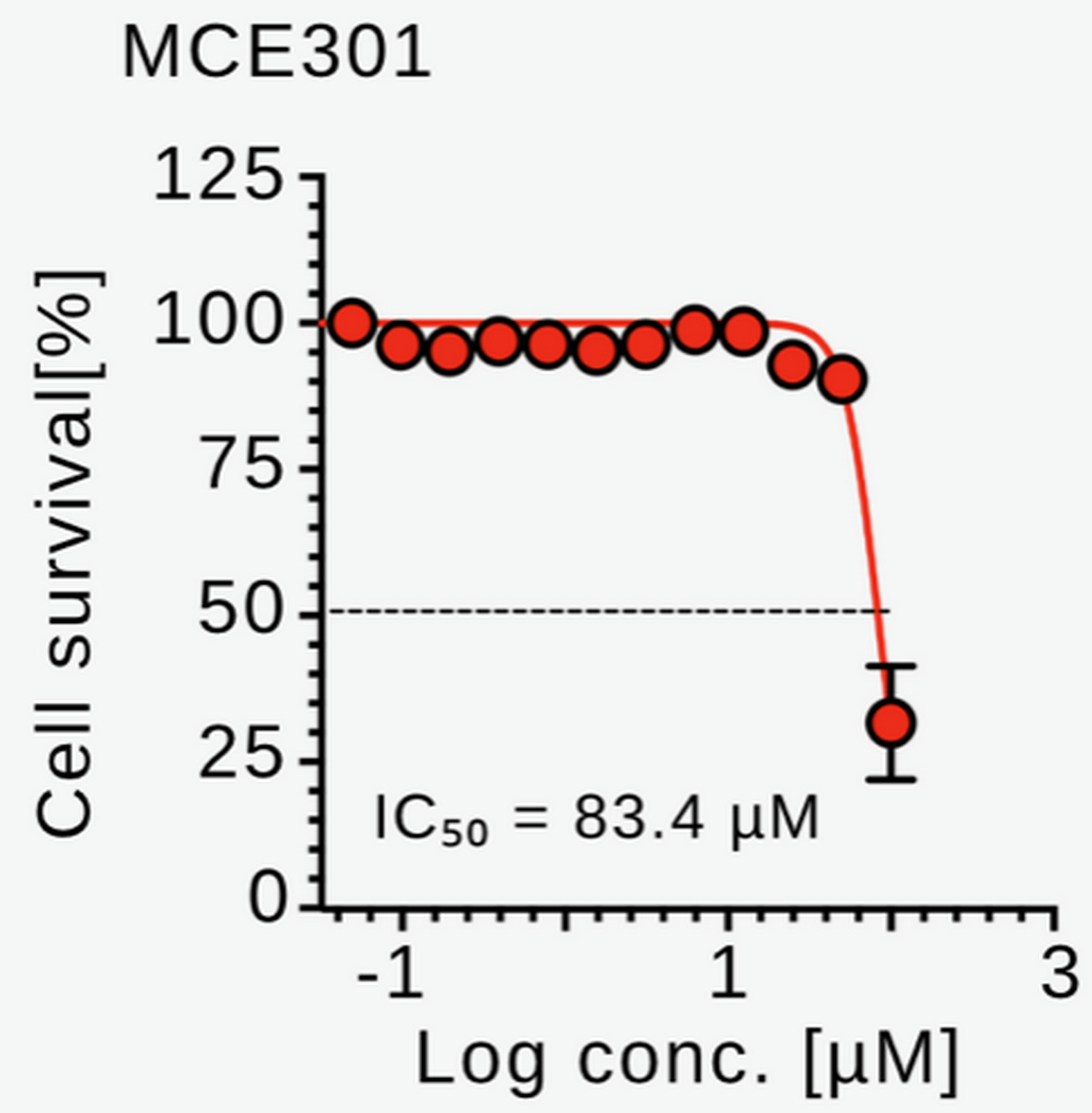

Supplement: S2 Fig — (TIF) [file pone.0334901.s003.tif]

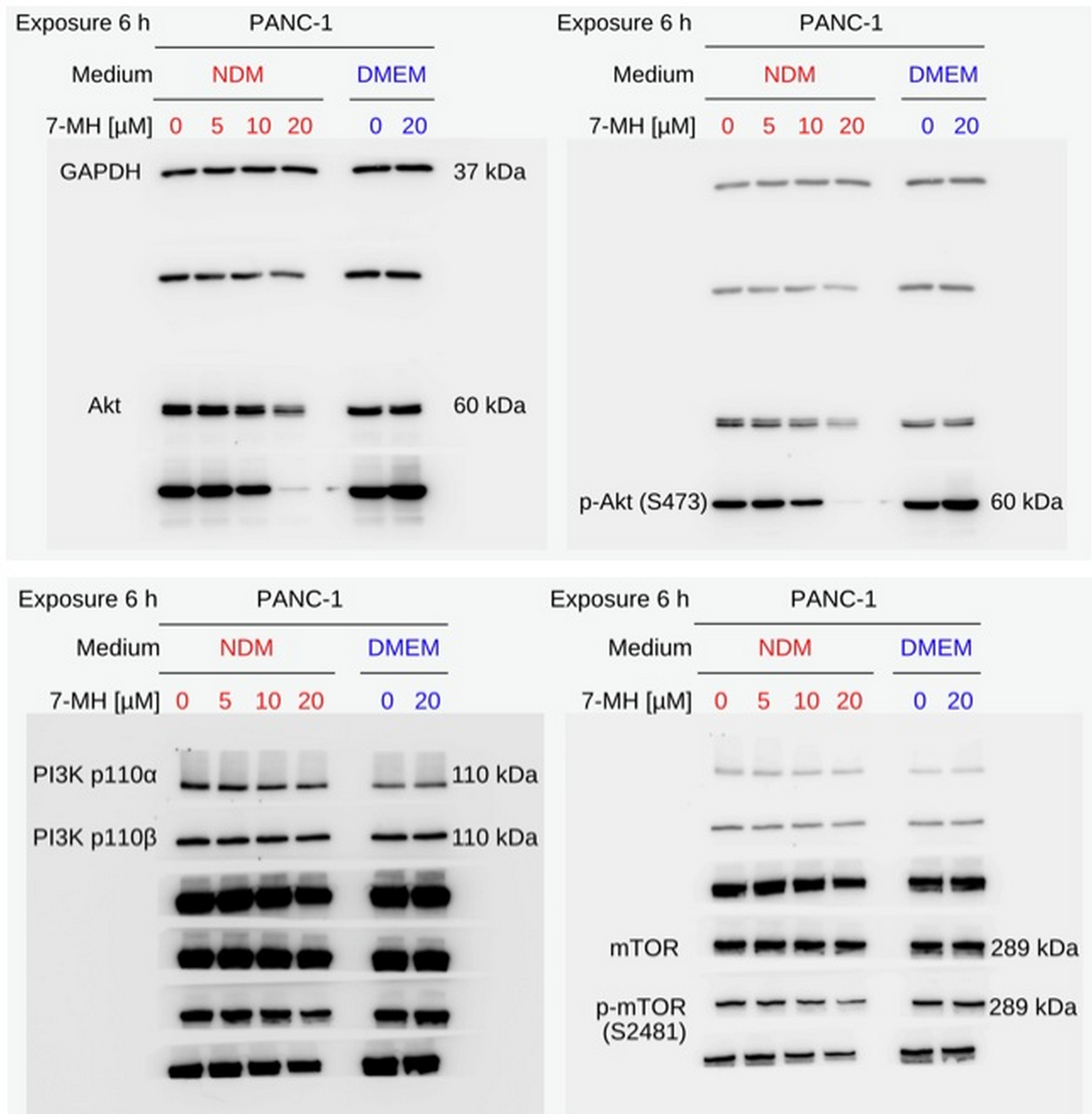

Supplement: S1 Data — (TIF) [file pone.0334901.s006.tif]
